# Leveraging AI in Postgraduate Medical Education for Rapid Skill Acquisition in Ultrasound-Guided Procedural Techniques

**DOI:** 10.3390/jimaging9100225

**Published:** 2023-10-16

**Authors:** Flora Wen Xin Xu, Amanda Min Hui Choo, Pamela Li Ming Ting, Shao Jin Ong, Deborah Khoo

**Affiliations:** National University Hospital, National University Health Systems, Singapore 119074, Singapore; flora.xu@mohh.com.sg (F.W.X.X.); amanda.choomh@mohh.com.sg (A.M.H.C.); tinglimingpamela@gmail.com (P.L.M.T.); deborah_khoo@nuhs.edu.sg (D.K.)

**Keywords:** ultrasound, training, nerve block, vascular access, quantitative feedback, deep learning, radiology, anaesthesia, simulation training

## Abstract

Ultrasound-guided techniques are increasingly prevalent and represent a gold standard of care. Skills such as needle visualisation, optimising the target image and directing the needle require deliberate practice. However, training opportunities remain limited by patient case load and safety considerations. Hence, there is a genuine and urgent need for trainees to attain accelerated skill acquisition in a time- and cost-efficient manner that minimises risk to patients. We propose a two-step solution: First, we have created an agar phantom model that simulates human tissue and structures like vessels and nerve bundles. Moreover, we have adopted deep learning techniques to provide trainees with live visualisation of target structures and automate assessment of their user speed and accuracy. Key structures like the needle tip, needle body, target blood vessels, and nerve bundles, are delineated in colour on the processed image, providing an opportunity for real-time guidance of needle positioning and target structure penetration. Quantitative feedback on user speed (time taken for target penetration), accuracy (penetration of correct target), and efficacy in needle positioning (percentage of frames where the full needle is visualised in a longitudinal plane) are also assessable using our model. Our program was able to demonstrate a sensitivity of 99.31%, specificity of 69.23%, accuracy of 91.33%, precision of 89.94%, recall of 99.31%, and F1 score of 0.94 in automated image labelling.

## 1. Introduction

Ultrasound-guided needle procedures are now prevalent in various medical specialties, such as interventional radiology, anaesthesia and musculoskeletal medicine. Directing a needle to its target under ultrasound guidance requires the healthcare practitioner to deftly keep the needle in a narrow ultrasound beam only millimetres wide. Failure to visualise the needle-tip throughout the procedure is a common error [1] which risks serious injury to nerves, blood vessels, and vital organs [2,3]. Traditionally, physicians acquire these skills through extensive hands-on training and mentorship. This conventional training approach of “see one, do one, teach one” on real patients risks patient morbidity. Therefore, there is great potential in exploring innovative training methods that can improve and accelerate skills acquisition.

Over the past decade, there have been tremendous developments in the field of ‘computer vision’, which includes object classification (identifying the type of object), localisation (identifying the location of the object), and detection (identifying both type and location of object). Deep learning (DL), a subset of machine learning (ML), has gained significant traction in computer vision applications. Particularly in the field of medical imaging, DL has proven useful for its ability to extract complex patterns and features from large datasets [4].

Detectron2, developed by Facebook AI Research (FAIR), serves as the successor to Detectron and the MaskRCNN Benchmark [5]. This deep learning library is recognised for delivering cutting-edge outcomes in object detection, image segmentation and other visual recognition tasks [4,6]. The platform is now implemented in PyTorch. Detectron2 encompasses high-quality implementations of various object detection methodologies, including Faster R-CNN, Mask R-CNN, RetinaNet, DensePose, Cascade R-CNN, Panoptic FPN, and TensorMask [7]. It offers support for three distinct types of segmentation: semantic segmentation, instance segmentation, and panoptic segmentation [8]. Panoptic segmentation aims to establish a comprehensive framework that bridges the gap between instance and semantic segmentation [9,10]. Its advanced algorithms and pre-trained models make it a powerful tool for medical image analysis. By leveraging DL frameworks like Detectron2, researchers and clinicians can enhance the training and assessment of ultrasound-guided needle procedures.

First, it enables the development of computer vision-based systems that can automatically detect and track the needle in real-time during practice sessions. This automated feedback system can provide immediate and objective assessments of needle placement accuracy, allowing trainees to evaluate and improve their technique.

Furthermore, DL models trained on large datasets of annotated ultrasound images can assist trainees in recognising critical structures, such as nerves or blood vessels, and avoiding potential complications during needle insertion [6,11]. By leveraging the capabilities of Detectron2, these models can provide real-time guidance and decision support, augmenting the trainee’s understanding of ultrasound anatomy.

In addition, DL can facilitate performance assessment and competency evaluation in ultrasound-guided needle procedures. Such models can provide quantitative metrics for assessing procedural skills, such as accuracy, precision, and efficiency. This objective evaluation can help identify areas for improvement and tailor individualised training programs to enhance trainee competence.

In this paper, we explore the use of Detectron2 and deep learning techniques in the context of ultrasound-guided needle procedures. We investigate the development of computer vision-based systems for real-time needle detection and tracking, as well as the integration of deep learning models for anatomical recognition and procedural guidance. Furthermore, we explore the potential of deep learning algorithms in assessing trainee performance and providing objective feedback in phantom-based training scenarios.

Through enhanced visualisation, real-time guidance, and automated performance assessment, we anticipate that deep learning-based approaches can accelerate the acquisition of skills, promote patient safety, and optimise the overall proficiency of healthcare professionals in ultrasound-guided interventions.

## 2. Materials and Methods

### 2.1. Creation of Agar-Based Ultrasound Phantom Model

**Figure 1:**
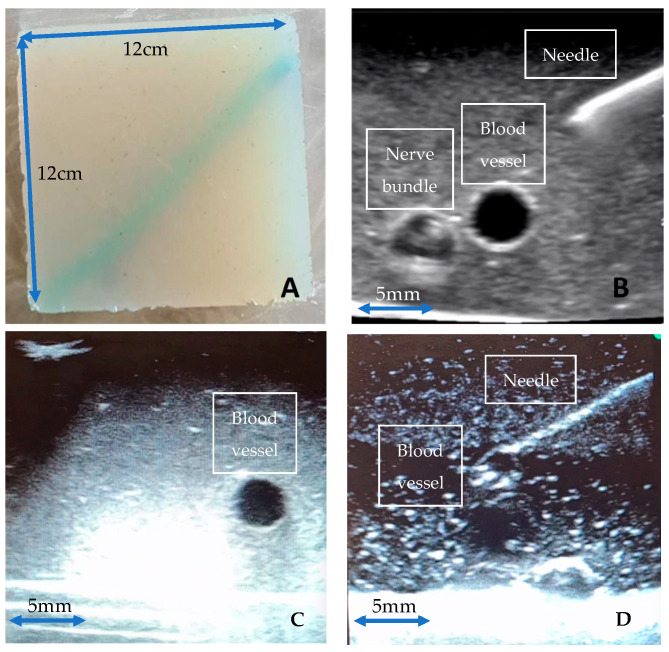
(**A**): 12 cm × 12 cm Agar phantom model; (**B**–**D**): Ultrasound images of Agar models (**A**–**C**), respectively, with echogenic needle, simulated vessels, and neurovascular bundles viewed on Butterfly iQ handheld probe.

We have created non-gelatine, food grade ingredient-based ultrasound phantom models that are high fidelity, inexpensive, replicable, and durable (Figure 1). Vessels were simulated using water-filled rubber sculpture balloons (30 cm in length and 0.6 cm in diameter) and nerve bundles were simulated using nylon 6/6 cable tie strips (each strip measuring 25 cm × 0.5 cm) enclosed within the water-filled sculpture balloons. Initial testing involved extensive experimentation with nearly 30 different combinations of agar [12], septanol [13], psyllium [14], and Konnyaku [15], amongst other materials. Three different final gel phantoms were selected for the training of our deep learning model: Phantom A. Agar 2.5% with septanol and psyllium husk (12.5 g of agar powder + 1 tsp of psyllium husk powder + 1 tbsp of septanol in 500 mL of water); Phantom B. Agar 2.5% with septanol; and Phantom C. “Smelleze” reconstituted fluid solidifier granules (15 mL of Smelleze [16] brand fluid solidifier and deodoriser granules dissolved in 500 mL of water). These phantoms were selected based on various factors including cost and ease of access, replicability, durability, and image texture, and are shown in Figure 1A–D below.

### 2.2. Creation of AI Model

Deep learning techniques were employed to automate needle tip detection on ultrasound images and successful penetration into blood vessels and nerve bundles. Our program utilised Detectron2 [17], an open access object detection and segmentation library developed by Facebook AI Research (FAIR) [5].

**Figure 2:**
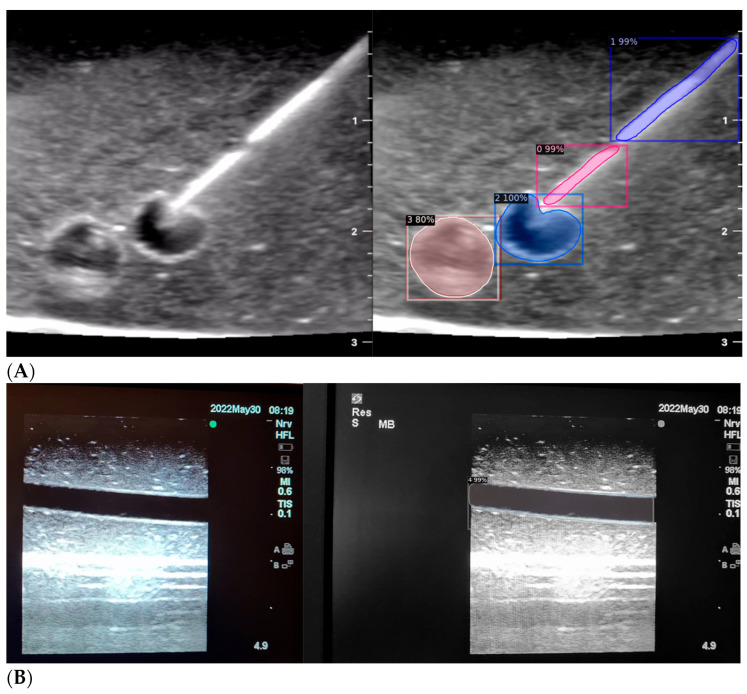
(**A**,**B**) AI-based program identifying needle tip (pink), needle shaft (purple), blood vessel (blue), and nerve (red) in ultrasound images.

It was written in Python and powered by the Caffe2 deep learning framework [18]. Frame-by-frame images were labelled using “labelme” software (“0” = needle tip, “1” = needle shaft, “2” = transverse section of blood vessel, “3” = transverse section of nerve). Following a randomised test–train split, our program was first trained on 186 frame-by-frame labelled images of needle entry in different agar-based models, then tested on a separate set of 196 unlabelled images from the same mediums (Figure 2).

**Figure 3:**
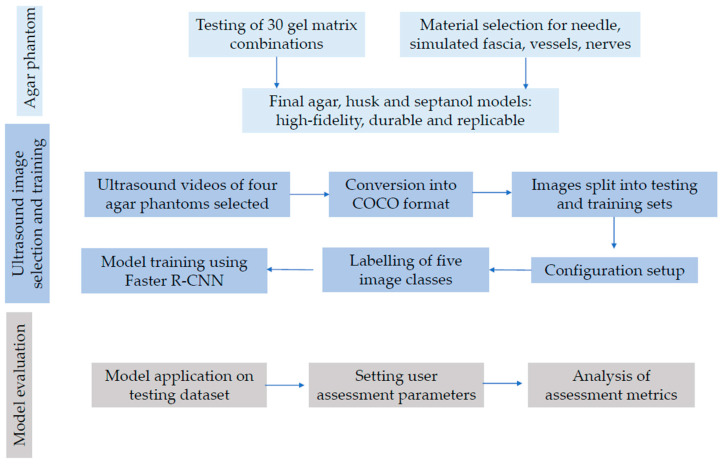
Process diagram of agar model creation and image segmentation framework.

Our multistep method is illustrated in a flow chart as follows (Figure 3). The process can be segmented into three stages, namely image selection and platform introduction; training; and model application and assessment.

The first stage involves preparing the image set for introduction to the platform, including the crucial step of dividing the set into training and test subsets [19]. This ensures proper utilisation of the data. The second stage encompasses setting up the platform and adjusting configuration values before entering the training phase [20]. It includes configuring the platform and fine-tuning the configuration parameters. The training process itself takes place within this stage. Finally, the third stage entails testing the trained model on the designated test set, evaluating various metrics like sensitivity and specificity to assess model performance [21]. The results in turn determine whether to conclude the training and testing process or to further modify training values for model performance enhancement.

#### 2.2.1. Detectron2 and Common Objects in Context (COCO) Format

The training process involved several key steps, starting with the preparation and annotation of a diverse dataset of ultrasound images captured from our phantoms. These images encompass a wide range of anatomical variations, planes of imaging (transverse, longitudinal, and oblique cuts of nerves and vessels), needle trajectories (in-plane and out-of-plane approaches), and tissue characteristics. A range of sonographic echogenicities is provided by the phantoms depending on the constituent ingredients (different agar combinations versus Smelleze granules), the concentration of the constituent ingredient (2.5% vs. 5%), and the addition or omission of psyllium husk. These variations in the phantoms enhance the model’s generalisability to the anatomical variability inherent in diverse patient populations. Manual annotation of the dataset was performed by one author (F.W.X.X), delineating critical structures, such as blood vessels, nerves, and target regions for needle placement. The annotations serve as ground truth labels to train the deep learning model.

Next, the annotated dataset was organised and formatted in the Common Objects in Context (COCO) format, which is the standard input format supported by Detectron2. We adopted a JSON-based image file system, enabling the labelling of objects of interest. This file structure encompasses three components: (i) Images, (ii) Categories, and (iii) Annotations [22]. Data is derived from binary masks or ground truth images where the original image is represented in black and the four marked classes (0 = needle tip, 1 = needle body, 2 = blood vessel, 3 = nerve bundle) are represented in differing colours in each frame. The “Images” component connects each image file with a unique identifier and includes information about its dimensions. The “Categories” component allows for the registration of each class or object category that belongs to a group of objects. Lastly, the “Annotations” component comprises an object identifier, its associated category, information on whether it is a group of objects or not, and a series of coordinate pairs that form a polygon outlining the region occupied by the object. Additionally, the annotations include the total area covered by the object and the coordinates defining its bounding box.

#### 2.2.2. Train–Test Split

To achieve a more diverse training and testing dataset, we incorporated ultrasound videos of three distinct gel phantoms (the constituents of which were detailed previously) namely: A. Agar 2.5% with septanol and psyllium husk, B Agar 2.5%with septanol, and C. “Smelleze” reconstituted fluid solidifier granules.

Each phantom model inherently possesses unique characteristics, such as distinct tissue densities, scattering properties, and acoustic responses, which result in varying ultrasound image appearances. By including images from multiple phantom models of different constituents, the dataset becomes more balanced and representative of a broader range of anatomical structures and scenarios. By encompassing this rich variety of scenarios, our approach mitigates the risk of over-emphasising specific structures that may dominate the dataset if only a single agar phantom were used. This ensures that the AI model gains a robust understanding of ultrasound image segmentation and becomes more adept at accurately identifying and analysing different anatomical features in various applications.

From the image pool, 186 were chosen for training purposes, while 196 were selected for testing. The train–test split was randomised using an automated random number generator. Four object classes (needle tip, needle body, blood vessel, and nerve bundle) were delineated in each image and their annotations were then verified by a second author.

#### 2.2.3. Configuration Setup for Training

The Detectron2 library provides a Python-based interface that allows customisation of the training process. Users can define and modify the configuration parameters to adapt the model to specific requirements. Our key parameter settings include the following:

(i) BASE LR: Learning rate was set to 0.00025;
(ii) MAX ITER: Maximum number of iterations for the training phase was set to 20,000;
(iii) MODEL.ROI HEADS.NUM CLASSES: Number of different object classes contained in the dataset was set to 4 (0 = needle tip, 1 = needle body, 2 = blood vessel, 3 = nerve bundle).

#### 2.2.4. Training

During this stage, the implementation of Detectron2 was carried out following the installation, configuration, and training guidelines provided in the official Detectron2 documentation.

Faster R-CNN, the model employed in our approach, consists of two interconnected modules that collaborate to accomplish different tasks [23]. The first module, known as the Region Proposal Network (RPN), is responsible for proposing regions of interest in the image. The RPN generates bounding boxes and assigns objective scores, indicating the probability of a region belonging to a particular class [23]. This is achieved through a fully convolutional network, which iterates over the image using a sliding-window approach. Each window is fed into a convolutional network with a fixed size, followed by two additional layers: the box-regression layer and the box-classification layer [24].

Around each window, anchors are positioned to capture variations in scale and aspect ratio. The number of anchors per image is typically determined by the formula width × height and guided by the scale markers on the side of each ultrasound image demarcating the image dimensions in centimetres (Figure 2). Anchors serve as reference points for region features without necessarily encompassing the entire region. This simplifies the subsequent processing in the Faster R-CNN module [23]. To reduce the number of region proposals, a non-maximum suppression method is applied, considering the scores assigned to each region [25]. This method eliminates redundant proposals by keeping only the highest-scoring regions in overlapping areas [25].

By employing this two-module architecture and incorporating region proposal generation and non-maximum suppression techniques, the Faster R-CNN model achieves accurate region localization and class detection in the ultrasound images of agar phantoms [23].

## 3. Results

### 3.1. Model Application and Assessment

**Figure 4:**
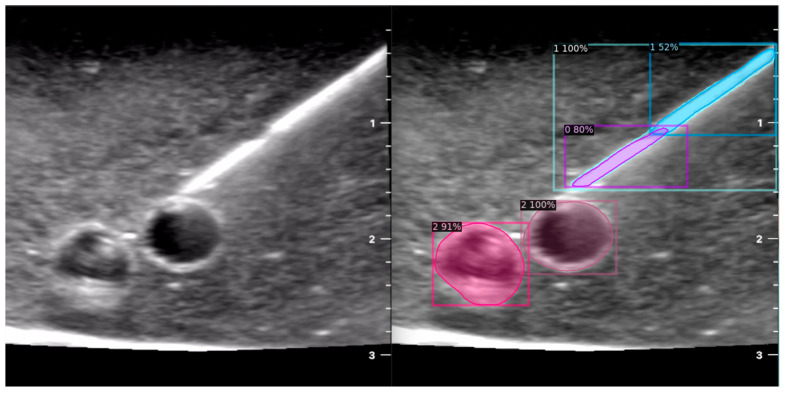
Model inference in Phantom A (Agar 2.5% with septanol and psyllium husk).

**Figure 5:**
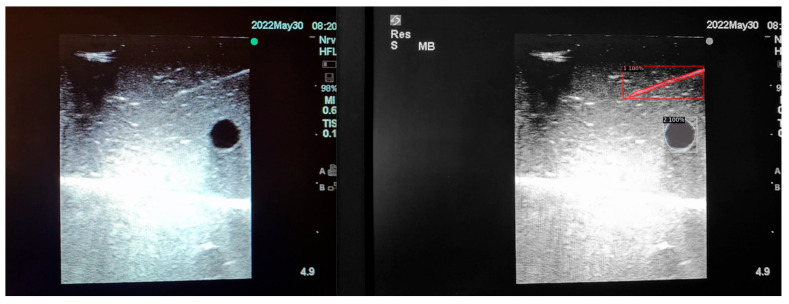
Model inference in Phantom B (Agar 2.5% with septanol).

When the model is applied, the original ultrasound image is overlaid with a new layer that presents the detection results, offering insights into predicted target areas (Figure 4 and Figure 5).

**Figure 6:**
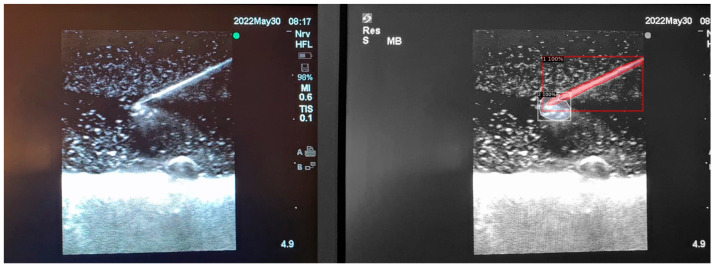
Model inference in Phantom C (Smelleze).

The segmentation area represents the region identified by the model as each object class (needle tip, needle body, blood vessel, or nerve bundle). This area is visually highlighted, allowing for a clear understanding of what structure is of interest. To provide additional context, a bounding box in the shape of a square is placed around the segmentation area, enclosing the detected object and providing a concise representation of its location. These are illustrated in Figure 4, Figure 5 and Figure 6, with original images on the left and overlayed images on the right for our three different phantom models.

To facilitate easy differentiation between different objects, Detectron2 generates a detection name based on the metadata of the COCO file. This name provides information about the specific object type, enabling the effective categorisation and analysis of different structures.

In order to assess the confidence level of the model’s predictions, a confidence percentage is assigned for each object class labelled (Figure 4, Figure 5 and Figure 6). This percentage represents the probability of a true positive detection by the model. It quantifies the model’s level of certainty regarding the accuracy of its prediction for each detected defect.

By incorporating these visual and numerical elements in the results, our approach using Detectron2 provides a comprehensive and informative output that aids in defect identification, localisation, and confidence assessment during ultrasound-guided procedures in simulation phantoms.

### 3.2. Assessment Metrics

The program’s effectiveness is assessed using sensitivity, specificity, precision, accuracy, and F1 scoring metrics [26,27]. These evaluation measures are widely employed in medical diagnostics and are defined as follows:

Sensitivity represents the proportion of true positive cases compared to the combined total of true positive and false negative cases [27]. Specificity, on the other hand, denotes the ratio of true negative cases to the sum of true negative and false positive cases [27]. Accuracy is the ratio of the sum of true positive and true negative cases to the overall sample [27].

In the context of Detectron2, the F1 score can be computed by comparing the model’s predicted bounding boxes or segmentations with the ground truth annotations [28]. Precision represents the proportion of true positive predictions among all positive predictions, while recall represents the proportion of true positive predictions among all actual positive instances [29]. High precision ensures that the model has fewer false positives, while high recall indicates that the model is successful in capturing most of the relevant structures in the images. By combining precision and recall using the harmonic mean, the F1 score provides a single value that indicates the overall performance of the object detection model [28].

### 3.3. Analysis of Sensitivity, Specificity, and Accuracy

Our image segmentation program has achieved remarkable results, with a prediction factor exceeding 95% in identifying most object classes, especially needle and vessel structures. The prediction factor displayed represents the model’s confidence that the labelled object belongs to a certain object class. However, in a select group of low-quality images, our model displays a low prediction factor, enabling the user to decipher that the labelling may not be accurate in such instances.

We evaluated our image segmentation model’s reliability using key metrics as follows (Table 1):

Furthermore, the model achieved an impressive F1 score of 0.94, demonstrating its exceptional ability to correctly identify both positive and negative examples in the ultrasound images. This is comparable to other studies applying DL models on gelatine models (F1 score of 0.95) [30] and human breast tissue (F1 scores ranging 0.83–0.87) [31]. These results highlight the effectiveness and reliability of our image segmentation model in most cases, making it a valuable tool for accurately delineating structures of interest in ultrasound images. Nonetheless, we acknowledge that there are limitations, particularly when dealing with low-quality images, where the accuracy may be affected. Continuous monitoring and improvement will be vital to ensure the model’s robustness and practical application in varied simulation phantoms.

### 3.4. Inaccuracies in Distorted Images

**Figure 7:**
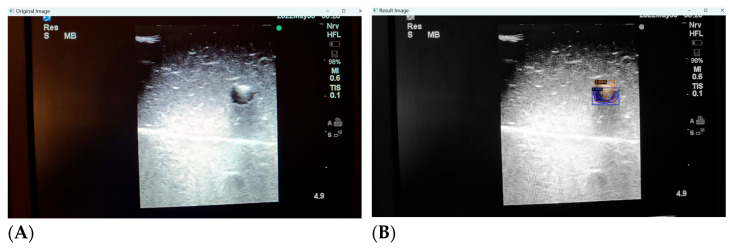
(**A**,**B**) Poor image quality leading to unclear identification of target structures.

While we continue to expand our training image pool and refine the deep learning process, we have identified a few key shortcomings with the automated detection of distorted or low quality images. Low quality ultrasound images can be defined as images with unclearly visualised structures (partially occluded or distorted vessels or needles, lack of clear delineation between structure and background) or those with noisy backgrounds (hyper or hypoechogenic depending on probe angulation, gain and scale settings, type of phantom model). Our deep learning algorithm is still able to identify the presence of the target structures with striking accuracy, but can mislabel surrounding noise as additional structures (Figure 7A,B).

**Figure 8:**
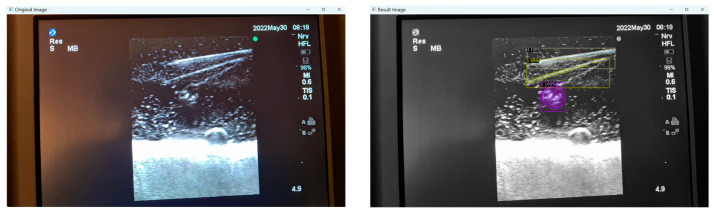
Original and result images of falsely identified needle.

Moreover, false positives can arise where structures that are not present are falsely identified as present. This can occur in certain mediums like Phantom C (Smelleze), where the echogenic needle track mark (overlayed in yellow) left behind is misidentified as a needle alongside the actual needle (overlayed in grey) as shown in Figure 8.

### 3.5. User Assessment

**Figure 9:**
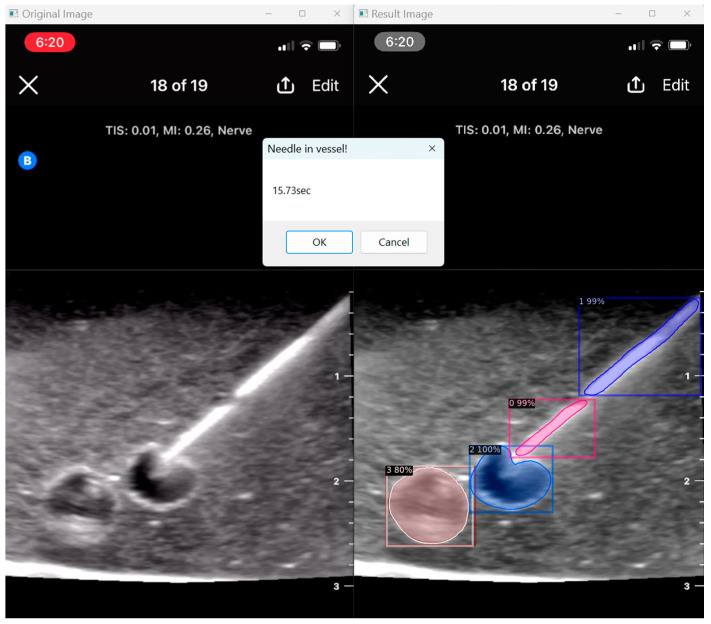
User speed assessment.

Our program presents an opportunity to automate ultrasound skill assessment over time, testing parameters like user speed (time taken for target structure penetration; Figure 9) and accuracy. With the automated segmentation of needle tip (item class 0) apart from needle body (item class 1), our program has the capacity to assess the percentage of frames for which a user keeps the full needle (including the tip) in view during their procedure. The capacities of AI in further assessment are vast, and possible features that can be expounded on include testing probe positioning and needle angulation.

## 4. Discussion

Detectron2 has proven to be a valuable tool for ultrasound image detection in agar phantoms. By leveraging the power of deep learning and the capabilities of the Detectron2 framework, the accurate and efficient detection of ultrasound images has been achieved. The use of agar phantoms provides a controlled and realistic environment for training and testing the model, simulating real-world ultrasound-guided procedures [32].

The results obtained through our implementation of Detectron2 have demonstrated the ability to accurately segment and detect target structures in ultrasound images. The segmentation area and bounding box generated by the model provide precise localisation of the needle parts, vessels, and nerves. The detection names assigned to these objects allow for easy differentiation and categorization of different types of object classes.

Furthermore, the confidence percentage associated with each detection provides a measure of the model’s certainty in its predictions. This information can be crucial in determining the reliability of the detected structures and guiding further decision-making in ultrasound-guided procedures.

There are several advantages to applying deep learning to the recognition of medical images. In clinical practice, targets viewed on ultrasound may have subtle variations in shape, orientation, or colour [33,34]. Occasionally, the target may be partially occluded or deformed (e.g., tenting of a vessel by an echogenic needle). The DL model is able to tolerate these deformations in the object and still identify the target with a high confidence percentage. Furthermore, the program allows for a high noise level, which is particularly suitable for ultrasound-guided procedures, where there may be significant variability in the users’ skill, the intrinsic composition of subjects’ tissue, and ultrasound systems’ image reconstruction algorithms resulting in the variable quality of ultrasound images [33,34]. The program can process the images at a reasonable recognition speed of 200 ms per a 1000 × 1000 pixel image. Being able to provide near-instant feedback to the user is crucial to ensure the procedure is executed safely, and performance feedback is received in a timely manner.

Using DL, users can obtain quantitative feedback, including information on the percentage of time the needle tip is kept in view, and the time taken for the target to be reached. This quantifiable assessment is an objective measure of performance that complements qualitative feedback and drives rapid improvement. Potentially, the trainee can use this to evaluate their own technique in the absence of a trainer, facilitating repeated, independent practice and self-directed learning in a low-risk setting requiring minimal costs and manpower. This is of relevance in our increasingly time-compressed and stretched healthcare system, which grapples with ensuring patient safety while training healthcare practitioners to a high standard.

Currently, the program has been trained on images obtained from a phantom agar model, which may display significant variation from images obtained from human subjects. However, the variation seen in images from the phantom model can be a reliable representation of the challenges and complexities encountered in actual ultrasound-guided procedures, thus enhancing the generalisability of the trained model.

In the model training stage, a large number of images are required so as to sufficiently train the model to generalise to clinical practice. Growing this program’s training set may help improve its performance in object detection. In addition, there may be a minor proportion of training set images that were of poor quality or inaccurately labelled. Object detection is still a supervised algorithm, which requires a trained user to label images correctly.

The significance of our work extends across different fields of medicine, where the accurate and efficient detection of ultrasound structures is of paramount importance for precise diagnosis and treatment. By employing Detectron2 in ultrasound image detection, healthcare professionals can benefit from improved accuracy, reduced human error, and enhanced efficiency in ultrasound-guided procedures.

For future directions, we may extend the use of the DL model to human subjects, such as human cadaveric specimens, healthy volunteers, or patients. Testing the DL model on ultrasound videos obtained from human subjects can enable accurate performance feedback in real clinical contexts. Users can be surveyed to provide a clearer validation of the utility of using this program for performance feedback. In the future, we aim to assess users’ performance in ultrasound-guided procedures before and after training on the agar model and obtaining quantitative feedback using the DL model. We anticipate that these assessments will reflect the usefulness of training on a phantom model for accelerating skills acquisition, by developing the psychomotor skills required for ultrasound-guided procedures.

## 5. Conclusions

Increasingly, ultrasound-guided techniques are seen as the gold standard in many procedures in anaesthesiology. However, trainees lack the opportunity and confidence to acquire these skills, with the current ‘see one, do one, teach one’ teaching practice. This pilot study explores the potential of harnessing AI in ultrasound skill training—with automated image segmentation, trainees are armed with a visual aid in localising structures and getting a sense of needle positioning in a 3D tissue space. Furthermore, our deep learning model can provide quantitative feedback on user speed and accuracy in needle tip visualisation. The multi-fold applications of AI empower self-directed learning, and present an exciting new mode of accelerating ultrasound mastery for trainees.

## Figures and Tables

**Table 1:** Model assessment metrics and results.

Metric	Definition	Results on Model
True positive	Correct identification of presence of needle, needle tip, and blood vessel	143
True negative	Correct identification of absence of needle, needle tip, or blood vessel	36
False positive	Misidentification of needle track mark as needle Misidentification of needle tip as blood vessel	12 4
False negative	Missed identification of needle, needle tip, or vessel	1
Sensitivity	True positive rate = $\frac{True\;positive}{True\;positive+False\;negative}$	99.31%
Specificity	True negative rate = $\frac{True\;negative}{True\;negative+False\;positive}$	69.23%
Accuracy	$\frac{True\;positive+True\;negative}{True\;positive+True\;negative+False\;positive+False\;negative}$	91.33%
Precision	$\frac{True\;positive}{True\;positive+False\;positive}$	89.94%
Recall	$\frac{True\;positive}{True\;positive+False\;negative}$	99.31%
F1	$2\times \frac{1}{\frac{1}{Precision}+\frac{1}{Recall}}$	0.94

## Data Availability

The data presented in this study are available on request from the corresponding author.
